# Serum creatinine, genetic susceptibility, and the risk of osteoporosis and fracture: a prospective cohort study from the UK Biobank

**DOI:** 10.3389/fendo.2025.1727636

**Published:** 2026-01-20

**Authors:** Weijie He, Yu Zhou, Jiaxuan Ding, Jiale Jiang, Hang Zhang, Chonghui Hu, Qiongyan Liu, Honglin Gu, Huimou Chen

**Affiliations:** 1 Dongguan Hospital of Guangzhou University of Chinese Medicine, Dongguan, Guangdong, China; 2 Department of General Surgery, Qilu Hospital of Shandong University, Jinan, China; 3 Department of Radiation Oncology, Sun Yat-sen Memorial Hospital, Sun Yat-sen University, Guangzhou, Guangdong, China; 4 The First Clinical College of Changsha Medical University, Changsha, Hunan, China; 5 The Third Clinical College of Guangzhou Medical University, Guangzhou, China; 6 Department of Pancreas Center, Guangdong Provincial People’s Hospital (Guangdong Academy of Medical Sciences), Southern Medical University, Guangzhou, Guangdong, China; 7 Department of General Surgery, Guangdong Provincial People’s Hospital (Guangdong Academy of Medical Sciences), Southern Medical University, Guangzhou, Guangdong, China; 8 Phase I Clinical Trial Centre, Sun Yat-sen MemorialHospital, Sun Yat-sen University, Guangzhou, Guangdong, China; 9 Department of Spine Surgery, Guangdong Provincial People’s Hospital (Guangdong Academy of Medical Sciences), Southern Medical University, Guangzhou, Guangdong, China; 10 Department of Oncology, Sun Yat-sen Memorial Hospital, Sun Yat-sen University, Guangzhou, Guangdong, China

**Keywords:** fractures, osteoporosis, polygenic risk score, serum creatinine, UK Biobank

## Abstract

**Background:**

Osteoporosis imposes a substantial fracture burden, yet scalable and dynamic biomarkers for risk stratification remain limited. Serum creatinine—routinely assayed and capturing intertwined muscle and renal signals—has been little studied for incident osteoporosis or fractures.

**Methods:**

In the UK Biobank, covariate-adjusted Cox models and restricted cubic splines assessed associations of baseline creatinine with incident osteoporosis and fractures. Creatinine was modeled categorically (six strata; G4 [70–80 μmol/L] as reference) and continuously. The pre-defined analysis was stratified by sex and age (>65) as well as the three tertiles of the polygenic risk score for osteoporosis (PRS); the multiplicative interaction term examined the relationship between creatinine and PRS.

**Results:**

Creatinine showed a U-shaped association with osteoporosis, with a nadir near 80 μmol/L (overall P<0.001; nonlinearity P<0.001). In piecewise models, each 1-μmol/L increase corresponded to a 2.0% lower osteoporosis risk within 25–80 μmol/L (HR = 0.980, 95% CI 0.978–0.982) and a 1.1% higher risk within 80–130 μmol/L (HR = 1.011, 95% CI 1.006–1.017). For fractures, the dose–response was J-shaped around the same nadir: risk decreased by 1.0% per μmol in 25–80 μmol/L (HR = 0.990, 95% CI 0.988–0.992) and increased by 0.3% per μmol in 80–130 μmol/L (HR = 1.003, 95% CI 0.999–1.006). Sex-stratified patterns were directionally concordant. In PRS-stratified categorical analyses (vs G4), the low-creatinine stratum (G1) carried higher osteoporosis risk across tertiles (HRs 1.925, 1.811, and 1.592 for low, intermediate, and high PRS). Creatinine×PRS interactions were not significant (osteoporosis P = 0.132; fractures P = 0.210).

**Conclusions:**

Baseline serum creatinine was significantly and nonlinearly associated with incident osteoporosis (U-shaped pattern) and fractures (J-shaped pattern), with the lowest risk observed at approximately 80 μmol/L, and there was a notable sex difference (higher in males). These associations were independent of genetic susceptibility, suggesting that serum creatinine may serve as a low-cost adjunct marker for clinical risk stratification and surveillance of osteoporosis.

## Introduction

Characterized by reduced bone mass and microarchitectural deterioration that increase skeletal fragility and fracture risk (1), osteoporosis has become a major global public-health challenge. In 2019, an estimated 178 million new fractures occurred worldwide (2). In China, osteoporotic fractures are projected to reach 4.83 million by 2035 and approximately 5.99 million by 2050 (3). Despite the integration of dual-energy X-ray absorptiometry (DXA) and traditional risk factors (age, sex, smoking, body mass index(BMI), and others) into widely used tools, such as the WHO Fracture Risk Assessment Tool (FRAX) (4, 5), limitations in accessibility at the primary-care level, cost, and accuracy persist (6). A substantial proportion of older adults are affected by osteoporosis and its sequelae, including osteoporotic fractures (7). Accordingly, there is a compelling public-health need to develop inexpensive, readily accessible, and easily quantifiable biomarkers for early detection and dynamic risk stratification.

Creatinine is a metabolic byproduct of muscle metabolism and has traditionally been used as a surrogate marker for glomerular filtration rate (GFR) and kidney function. Recent epidemiological evidence indicates that serum creatinine reflects kidney function and, to a certain degree, also reflects the quantity and nutritional status of skeletal muscle (8, 9). Low creatinine levels are typically indicative of sarcopenia and/or malnutrition, with decreased muscle mass considered a significant risk factor for osteoporosis (10). Clinical and cohort studies further demonstrate that resistance exercise and nutritional optimization—designed to enhance muscle strength and mass—are associated with higher bone mass and a lower risk of fractures (11, 12). In contrast, elevated serum creatinine is commonly associated with chronic kidney disease (CKD). CKD-related mineral and bone disorders (CKD-MBD), through mechanisms such as calcium-phosphate imbalance, secondary hyperparathyroidism, vitamin D deficiency, and metabolic acidosis, contribute to disrupted bone metabolism and significantly increase fracture risk (13–15). However, to the best of our knowledge, there is currently no epidemiological study that directly evaluates the relationship between serum creatinine and the risk of osteoporosis and fractures.

In human genetics, large-scale genome-wide association studies (GWAS) have identified numerous single nucleotide polymorphisms (SNPs) associated with bone mineral density (BMD) (16), thereby advancing the development of polygenic risk scores (PRS). PRS quantifies genetic susceptibility and improves the accuracy of predicting osteoporosis risk (17–20). Beyond prediction, PRS is used as a stratification tool to assess whether the associations between creatinine and osteoporosis, as well as between creatinine and fracture incidence, remain consistent across different genetic backgrounds, thereby providing context for the physiological signals reflected by creatinine.

Thus, using data from more than half a million middle-aged and older participants in the UK Biobank, this study systematically evaluates the association between baseline serum creatinine and incident osteoporosis and fracture, assesses effect modification by polygenic risk, and quantifies the incremental predictive value of creatinine beyond conventional clinical predictors to inform pragmatic, easily implementable risk-assessment frameworks.

## Methods

### Study participants and data sources

This investigation was embedded within the prospective UK Biobank cohort, which enrolled more than 500,000 individuals aged 40–69 years between 2006 and 2010. At 22 assessment centers across England, Wales, and Scotland, participants completed touchscreen self-report questionnaires and underwent standardized physical measurements. Drawing on questionnaires, clinical assessments, and biospecimen collection, we compiled comprehensive biochemical data, designating baseline serum creatinine (µmol/L) as the primary exposure. Genotyping was conducted centrally by UK Biobank, and linked health-record data were used to ascertain longitudinal endpoints. Participants were included if serum creatinine, key covariates, and bone-related outcomes were available. Serum creatinine was measured in the UK Biobank central laboratory using an enzymatic method on a Beckman Coulter AU5800 platform, following UK Biobank biomarker quality procedures. We excluded individuals with a prior diagnosis of osteoporosis, osteoporotic fracture at baseline or participants who had withdrawn consent; those with missing creatinine data or PRS data; and those who lacked successful follow-up linkage. Written informed consent was obtained from all UK Biobank participants via a touchscreen interface. Ethical approvals were granted by the North West Multi-centre Research Ethics Committee, the National Information Governance Board for Health and Social Care in England and Wales, and Scotland’s Community Health Index Advisory Group (www.ukbiobank.ac.uk/ethics/). This analysis was conducted under application number 85224 using UK Biobank resources.

### Endpoints and covariates

Participants were followed from their first Assessment Centre visit until the earliest of death, loss to follow-up, or administrative censoring on 31 October 2022. During follow-up, incident osteoporosis and fractures were identified using International Classification of Diseases, 10th Revision (ICD-10)codes from linked health records, including hospital inpatient admissions and death registry records. Incident osteoporosis and fractures were defined as the first recorded diagnosis/event occurring after baseline (**Supplementary Table S2**). Case ascertainment was based on linked, multi-source health records, including death registrations, primary care records, and hospital admissions, as well as participant self-reports. Covariates included sociodemographic factors (age, sex, race/ethnicity, education, Townsend Deprivation Index) and lifestyle variables, including BMI, smoking status, alcohol use, adherence to physical activity guidelines, sleep profile, CKD status, and ambient fine particulate matter (PM2.5).

### Polygenic risk scores

UK Biobank generated imputed genotype data for 488,000 participants and performed standard genotyping quality control (QC) procedures (21, 22). Participants those with incongruent self-report and genetic sex were excluded from the study. Participants were included if they had genetic ancestry assignment information and met the QC standards (variant missing rate <2%). We obtained the PRS for participants with osteoporosis from the UK Biobank and standardized it within ethnic groups (mean ≈ 0, standard deviation ≈ 1) (23). The osteoporosis (estimated BMD) PRS was derived from meta-analyses of published genome-wide association studies (GWAS) and subsequently tuned in the UK Biobank (22). In participants of European ancestry, the PRS showed strong predictive performance: each 1-SD higher PRS was associated with a 0.427-SD higher estimated BMD, placing it among the top-performing scores across the quantitative traits evaluated. PRS were categorized into tertiles—low, intermediate, and high risk.

### Statistical analysis

Creatinine was categorized *a priori* into six groups (G1 <50; G2 50–60; G3 60–70; G4 70–80; G5 80–90; G6 ≥90 µmol/L), with G4 (70–80 µmol/L) as the reference group. For dose–response assessment, restricted cubic splines (RCS) were applied to creatinine in the Cox framework, adjusting for the prespecified covariates, and the overall P value and the P for non-linearity were reported to test the global effect and departures from linearity. Anchoring at the spline minimum, piecewise-linear Cox models were specified for the 25–80 µmol/L and 80–130 µmol/L ranges to compute HRs per 1-µmol/L increase within each interval. Multivariable categorical Cox models were then used to estimate HRs (95% CIs) for G1–G3, G5, and G6 relative to G4 was included to assess potential risk re-escalation at the upper creatinine range. Furthermore, we also conducted subgroup analyses based on age groups(>65), and evaluated the moderating effect of age on the outcome in the Cox model. Sex-stratified models were fitted in parallel. PRS-stratified Cox models were fitted across three PRS strata; in the full sample, we included an interaction term between creatinine categories and the PRS to obtain the P for interaction and evaluate effect modification. We used bootstrap resampling to quantify uncertainty in the estimated nadir (and/or piecewise slopes) of the creatinine–outcome associations. All models adjusted for age, sex, BMI, race/ethnicity, education, the Townsend Deprivation Index, physical activity, CKD status, smoking, and alcohol use frequency. All tests were two-sided with α=0.05. Statistical analyses were performed in R (version 4.5.0).

## Results

### Participants

**Figure 1:**
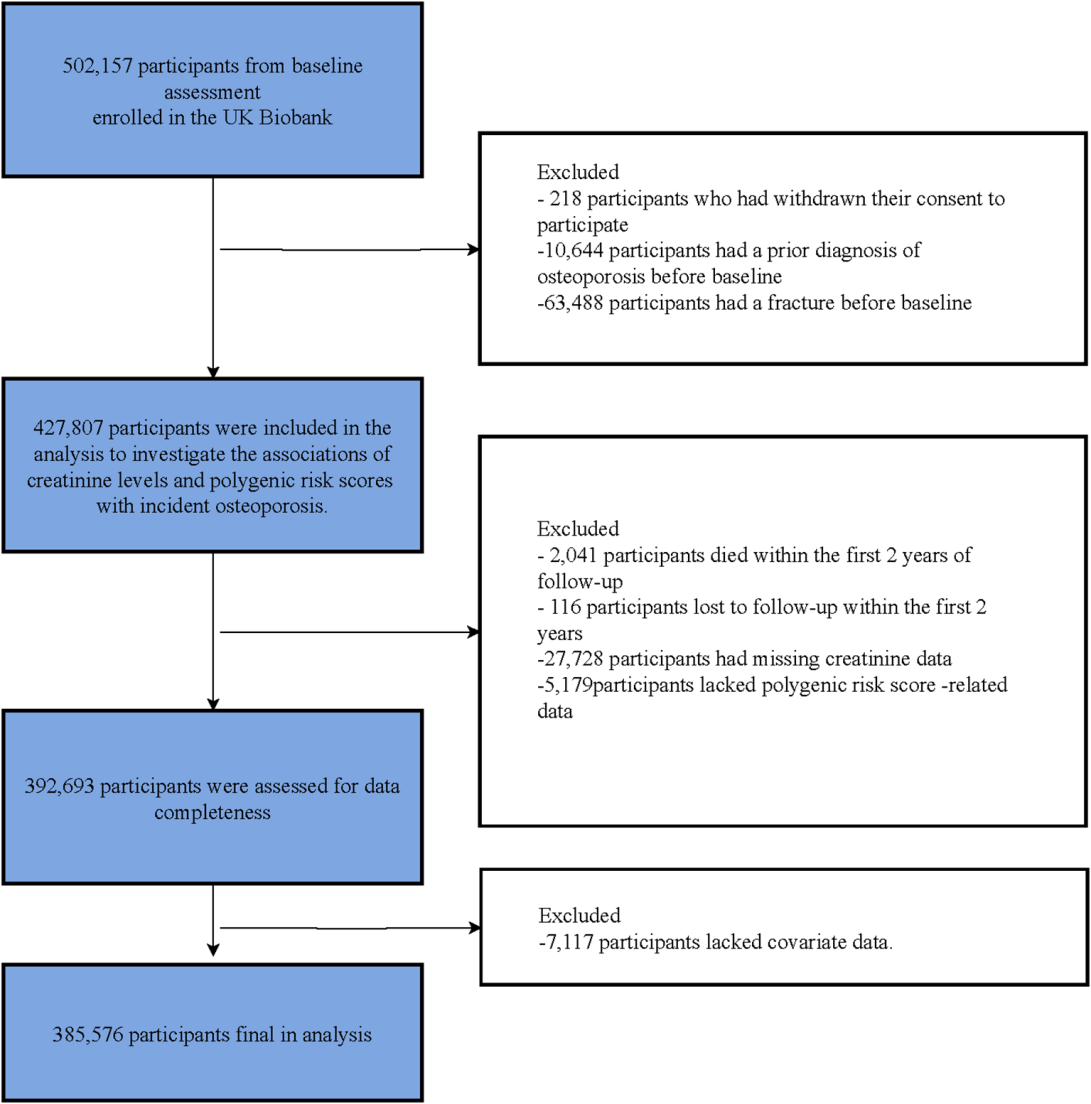
Flowchart of participants selection. Stepwise flowchart from the UK Biobank baseline sample (N = 502,157) through exclusions to the final analytic cohort (N = 385,576). Exclusions include withdrawn consent, prior osteoporosis or fracture, early death or loss to follow-up, missing creatinine, missing PRS, and missing covariates.

After screening for eligibility, 385,576 participants met the inclusion criteria and were included in the analysis cohort. **Figure 1** illustrates the sequential steps used to construct the analytic cohort. The baseline characteristics of the included participants are shown in **Table 1**. The mean age of participants with osteoporosis was 60.98 years (SD 6.33), whereas that of non-cases was 56.26 years (SD 8.10). Most new osteoporosis cases occurred in females (82.59%), with the majority being white (91.81%. Furthermore, 26,474 participants with fractures were included in the study, with the mean age of fracture cases being 58.54 years (SD 7.77). Female participants accounted for 46.46%, male participants for 53.54%, and 90.71% were white. Finally, the total number of participants was divided into six groups based on baseline serum creatinine: G1 (13,633), G2 (68,236), G3 (106,318), G4 (93,814), G5 (61,076), and G6 (42,499). Baseline characteristics stratified by creatinine groups are presented in **Supplementary Table S1**.

**Table 1:** Baseline characteristics of the study population stratified by fracture and osteoporosis.

	Non-fracture	Fracture	P	Non-osteoporosis	Osteoporosis	P
n	359102	26474		373041	12535	
Male (%)	166853 (46.46)	10104 (38.17)	<0.001	174775 (46.85)	2182 (17.41)	<0.001
Age (mean (SD))	56.26 (8.09)	58.54 (7.77)	<0.001	56.26 (8.10)	60.98 (6.33)	<0.001
White people (%)	325726 (90.71)	24445 (92.34)	<0.001	338663 (90.78)	11508 (91.81)	<0.001
Alcohol frequency			<0.001			<0.001
<3 times/week	174843 (48.69)	12563 (47.45)		181138 (48.56)	6268 (50.00)	
>=3 times/week	156687 (43.63)	11602 (43.82)		163545 (43.84)	4744 (37.85)	
Never/Other	27572 (7.68)	2309 (8.72)		28358 (7.60)	1523 (12.15)	
Smoking frequency			<0.001			<0.001
Current	36172 (10.07)	3023 (11.42)		37825 (10.14)	1370 (10.93)	
Never	198941 (55.40)	13795 (52.11)		206061 (55.24)	6675 (53.25)	
Previous	123989 (34.53)	9656 (36.47)		129155 (34.62)	4490 (35.82)	
BMI (%)			<0.001			<0.001
>30	87098 (24.25)	6302 (23.80)		91074 (24.41)	2326 (18.56)	
25-30	153949 (42.87)	10820 (40.87)		160293 (42.97)	4476 (35.71)	
Other	118055 (32.88)	9352 (35.33)		121674 (32.62)	5733 (45.74)	
Physical Meeting recommendation (%)	229559 (63.93)	16482 (62.26)	<0.001	238800 (64.01)	7241 (57.77)	<0.001
Qualification category (%)			<0.001			<0.001
High	118742 (33.07)	8079 (30.52)		123480 (33.10)	3341 (26.65)	
Intermediate	162158 (45.16)	11296 (42.67)		168331 (45.12)	5123 (40.87)	
Low	78202 (21.78)	7099 (26.81)		81230 (21.78)	4071 (32.48)	
CKD status	4074 (1.13)	431 (1.63)	<0.001	4270 (1.14)	235 (1.87)	<0.001
Townsend (mean (SD))	-1.39 (3.04)	-1.20 (3.13)	<0.001	-1.38 (3.04)	-1.17 (3.13)	<0.001
Sleep duration (mean (SD))	7.12 (1.23)	7.09 (1.34)	<0.001	7.12 (1.23)	7.06 (1.47)	<0.001
PM 2.5 (mean (SD))	9.98 (1.01)	10.00 (1.03)	<0.001	9.98 (1.01)	10.05 (1.03)	<0.001
Outcome Osteoporosis	8147 (2.27)	4388 (16.57)	<0.001	0 (0.00)	12535 (100.00)	<0.001
Outcome Fracture	0 (0.00)	26474 (100.00)	<0.001	22086 (5.92)	4388 (35.01)	<0.001

Data are presented as mean (standard deviation) or number (%);CKD: Chronic Kidney Disease; Townsend: Townsend deprivation index, a measure of socioeconomic status; PM 2.5: Particulate matter with a diameter of ≤2.5 micrometers; Because of rounding, percentage totals may not equal exactly 100%.

### Dose-response relationships between creatinine and risk of osteoporosis and fracture

**Figure 2:**
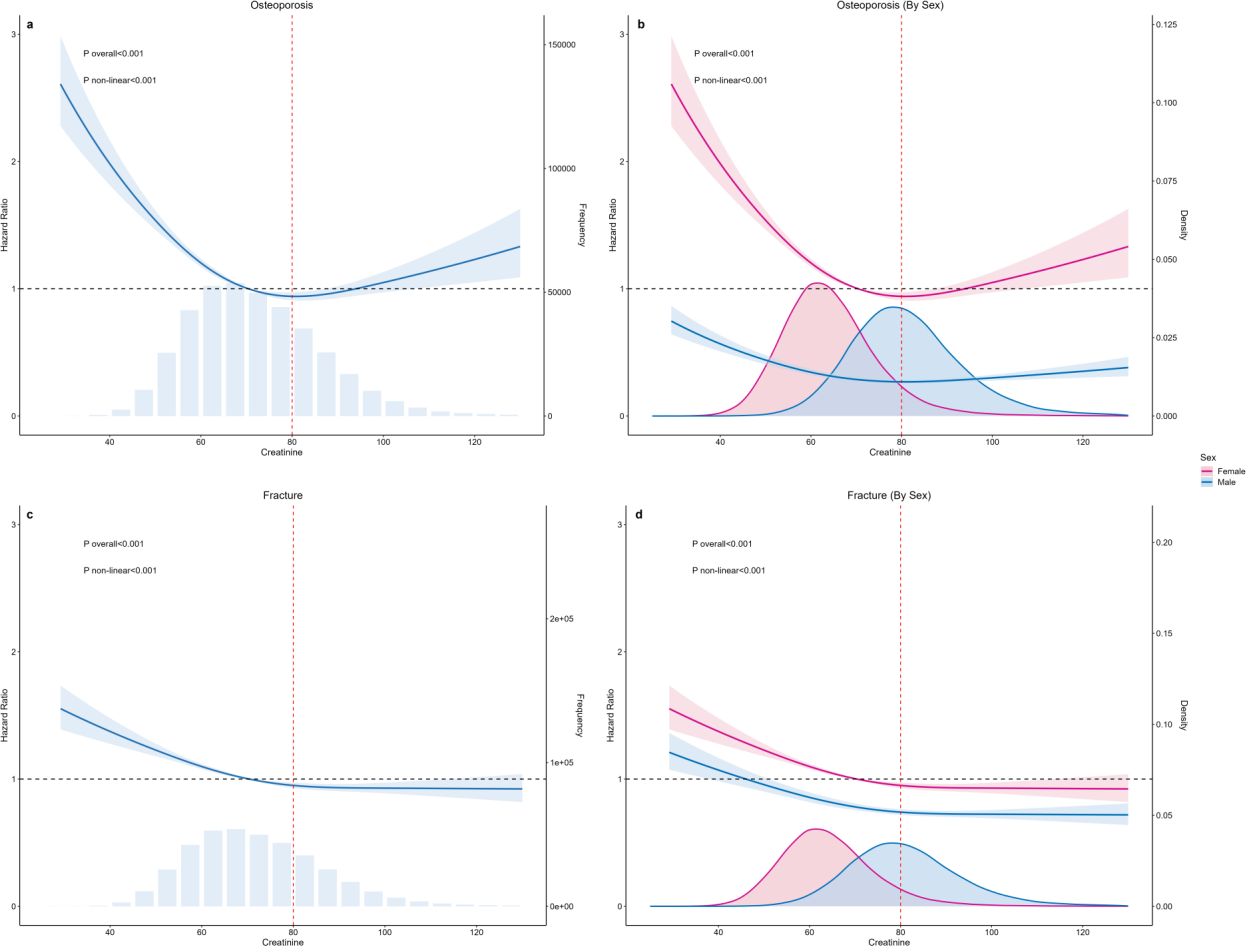
Nonlinear dose–response between serum creatinine and osteoporosis/fracture risk: spline curves for the overall cohort and by sex. **(A, C)** depict the nonlinear relationships of serum creatinine (µmol/L) with incident osteoporosis and incident fracture, respectively; **(B, D)** display the sex-specific counterparts. Curves represent spline-estimated hazard ratios (HRs), and the shading denotes 95% confidence intervals. he horizontal axis is creatinine concentration and the vertical axis is the HR; the black dashed line indicates the HR = 1 reference line. A red vertical dashed line indicates the ~80 µmol/L reference point corresponding to the risk nadir. The histograms beneath **(A, C)** depict creatinine distributions for the full sample, while overlaid kernel density estimates in Panels b and d show the distributions for females (pink) and males (blue). P overall and P non-linear indicate the P values for the overall association and for the nonlinearity test, respectively. Models were adjusted for prespecified covariates (age, BMI, ethnicity, education, Townsend deprivation index, physical activity, smoking, alcohol frequency, and ambient PM2.5, as appropriate). Restricted-cubic-spline curves show a U-shape for osteoporosis and a J-shape for fractures with a nadir near ~80 µmol/L. Overall, women, and men are shown separately; the vertical dashed line marks the 70–80 µmol/L reference window; 95% CIs widen at extremes. Overall and nonlinearity P < 0.001.

**Figure 3:**
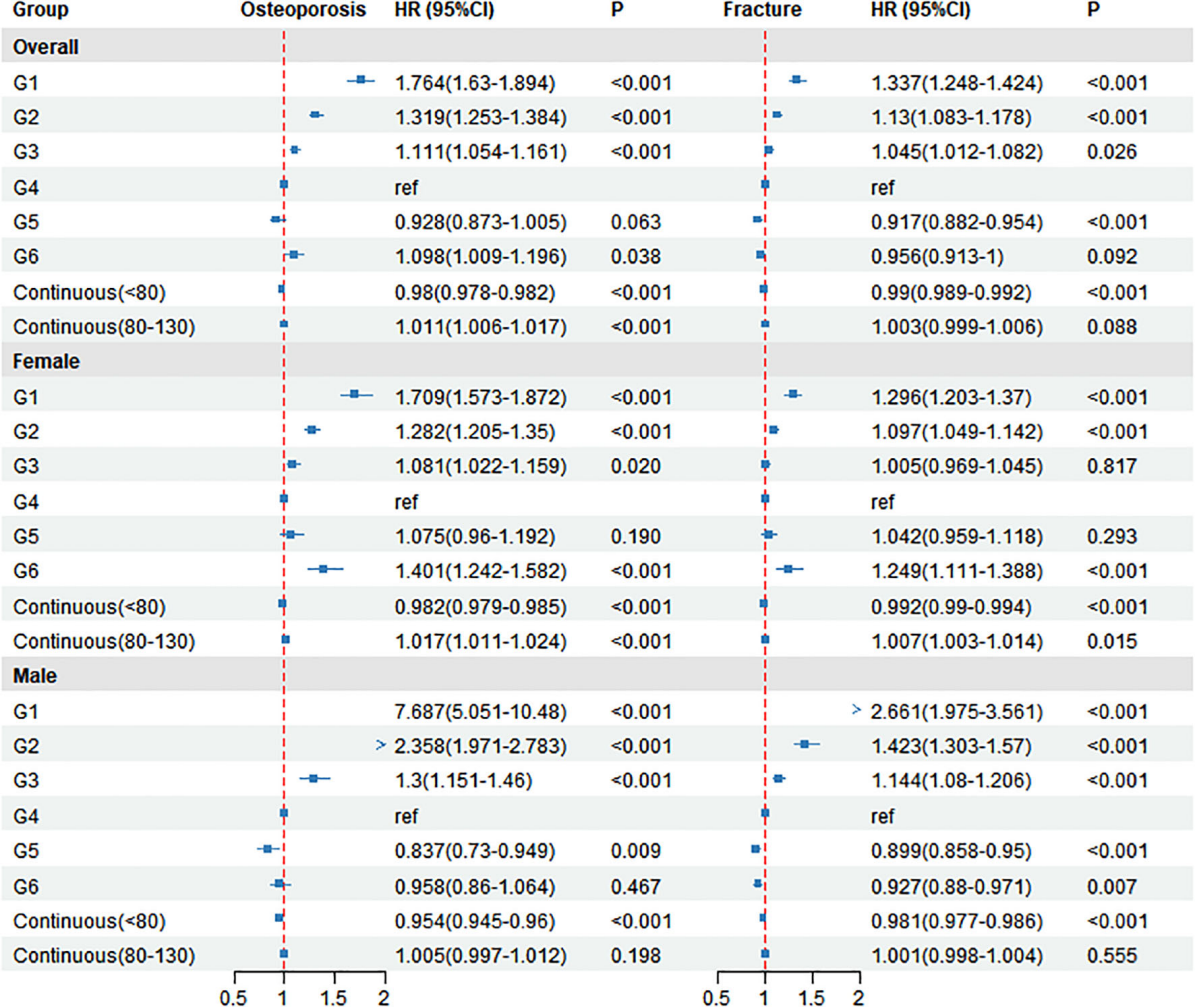
Association of creatinine categories and piece-wise continuous effects with incident osteoporosis: overall and by sex. HR: hazard ratio; CI: confidence interval ref: reference; G1: group 1; G2: group 2; G3: group 3; G4: group 4; G5: group 5; G6: group 6. Forest plot comparing creatinine groups G1–G3, G5, G6 to reference G4 for osteoporosis and fractures. Overall results: osteoporosis HRs are elevated at low creatinine (e.g., G1 = 1.761) and re-increase at the high end (G6 = 1.095), while fractures are similarly higher at low creatinine (G1 = 1.332) and lower in G5 (0.917); G6 for fractures is near null (0.956, P = 0.064). Sex-specific rows show larger effects in men (e.g., osteoporosis G1 = 7.704).

In the restricted cubic spline (RCS) model, **Figure 2** illustrates a U-shaped relationship between serum creatinine and incident osteoporosis (overall P < 0.001; nonlinear test P < 0.001), with the lowest risk occurring at approximately 80 µmol/L. Each 1 µmol/L increase in creatinine within the 25–80 µmol/L range is associated with a 2.0% reduction in risk (HR = 0.980, 95% CI 0.978–0.982) (**Figure 3**); in the 80–130 µmol/L range, risk increases by 1.1% (HR = 1.011, 95% CI 1.006–1.016). The sex-specific model reveals that in men, the risk decreases by 4.6% in the 25–80 µmol/L range (HR = 0.954, 95% CI 0.947–0.962, P < 0.001), with no significant change in the 80–130 µmol/L range (HR ≈ 1.005, P = 0.15); in women, the risk decreases by 1.8% in the 25–80 µmol/L range (HR = 0.982, 95% CI 0.980–0.985), and increases by 1.7% in the 80–130 µmol/L range (HR = 1.017, 95% CI 1.010–1.025), with both P < 0.001.

The same method was used to analyze incident fractures, with RCS analysis revealing a J-shaped curve (overall P < 0.001; nonlinear test P < 0.001), with the lowest risk point also around 80 µmol/L. The segmented linear model shows that in the 25–80 µmol/L range, each 1 µmol/L increase in creatinine is associated with a 1.0% reduction in fracture risk (HR = 0.990, 95% CI 0.988–0.992); in the 80–130 µmol/L range, the risk increases slightly by 0.3% (HR = 1.003, 95% CI 1.000–1.006, P = 0.064). The sex-stratified results show that in men, the risk decreases by 1.9% in the 25–80 µmol/L range (HR = 0.981, 95% CI 0.977–0.985), with no significant change in risk in the 80–130 µmol/L range (HR ≈ 1.001, P = 0.52); in women, the risk decreases by 0.8% in the 25–80 µmol/L range (HR = 0.992, 95% CI 0.990–0.994, P < 0.001), and increases by 0.7% in the 80–130 µmol/L range (HR = 1.007, 95% CI 1.001–1.014, P = 0.017).

### Risk of osteoporosis and fracture in multivariable Cox models

In the multivariate Cox regression model, serum creatinine levels were associated with incident osteoporosis and fractures (**Figure 3**). With the G4 group (70–80 µmol/L) as the reference, the hazard ratios (HRs, 95% CIs) for incident osteoporosis in the G1–G3 and G5–G6 groups were 1.764 (1.63–1.894), 1.319 (1.253–1.384), 1.111 (1.054–1.161), 0.928 (0.873–1.005), and 1.098 (1.009–1.196), with P < 0.001 for G1–G3, P = 0.063 for G5, and P = 0.038 for G6. Using 80 µmol/L as the cutoff for the piecewise continuous analysis, each 1 µmol/L increase in creatinine was associated with an HR of 0.98 (95% CI 0.978–0.982; P < 0.001) for creatinine <80 µmol/L, and an HR of 1.011 (95% CI 1.006–1.017; P < 0.001) for creatinine 80–130 µmol/L.

For incident fractures, the corresponding HRs (95% CIs) for G1–G3 and G5–G6 were 1.337 (1.248–1.424), 1.13 (1.083–1.178), 1.045 (1.012–1.082), 0.917 (0.882–0.954), and 0.956 (0.913–1.000), with P < 0.001 for G1, G2, and G5, P = 0.026 for G3, and P = 0.092 for G6. In the piecewise continuous analysis, each 1 µmol/L increase in creatinine corresponded to an HR of 0.99 (95% CI 0.989–0.992; P < 0.001) for creatinine <80 µmol/L and an HR of 1.003 (95% CI 0.999–1.006; P = 0.088) for creatinine 80–130 µmol/L.

Sex-stratified analyses showed similar patterns for osteoporosis: in women, HRs (95% CIs) were 1.709 (1.573–1.872), 1.282 (1.205–1.350), 1.081 (1.022–1.159), 1.075 (0.960–1.192), and 1.401 (1.242–1.582) for G1–G3 and G5–G6 (P < 0.001 for G1, G2, and G6; P = 0.020 for G3; P = 0.190 for G5), and the piecewise continuous HRs were 0.982 (0.979–0.985; P < 0.001) for <80 µmol/L and 1.017 (1.011–1.024; P < 0.001) for 80–130 µmol/L; in men, the corresponding HRs (95% CIs) were 7.687 (5.051–10.480), 2.358 (1.971–2.783), 1.3 (1.151–1.46), 0.837 (0.73–0.949), and 0.958 (0.86–1.064) (P < 0.001 for G1–G3; P = 0.009 for G5; P = 0.467 for G6), with piecewise continuous HRs of 0.954 (0.945–0.960; P < 0.001) for <80 µmol/L and 1.005 (0.997–1.012; P = 0.198) for 80–130 µmol/L. For fractures, women had HRs (95% CIs) of 1.296 (1.203–1.370), 1.097 (1.049–1.142), 1.005 (0.969–1.045), 1.042 (0.959–1.118), and 1.249 (1.111–1.388) for G1–G3 and G5–G6 (P < 0.001 for G1, G2, and G6; P = 0.817 for G3; P = 0.293 for G5), with piecewise continuous HRs of 0.992 (0.99–0.994; P < 0.001) for <80 µmol/L and 1.007 (1.003–1.014; P = 0.015) for 80–130 µmol/L; men had HRs (95% CIs) of 2.661 (1.975–3.561), 1.423 (1.303–1.570), 1.144 (1.08–1.206), 0.899 (0.858–0.950), and 0.927 (0.880–0.971) (P < 0.001 for G1–G3 and G5; P = 0.007 for G6), with piecewise continuous HRs of 0.981 (0.977–0.986; P < 0.001) for <80 µmol/L and 1.001 (0.998–1.004; P = 0.555) for 80–130 µmol/L.

### The risk of osteoporosis and fracture in different osteoporosis PRS subgroups

**Figure 4:**
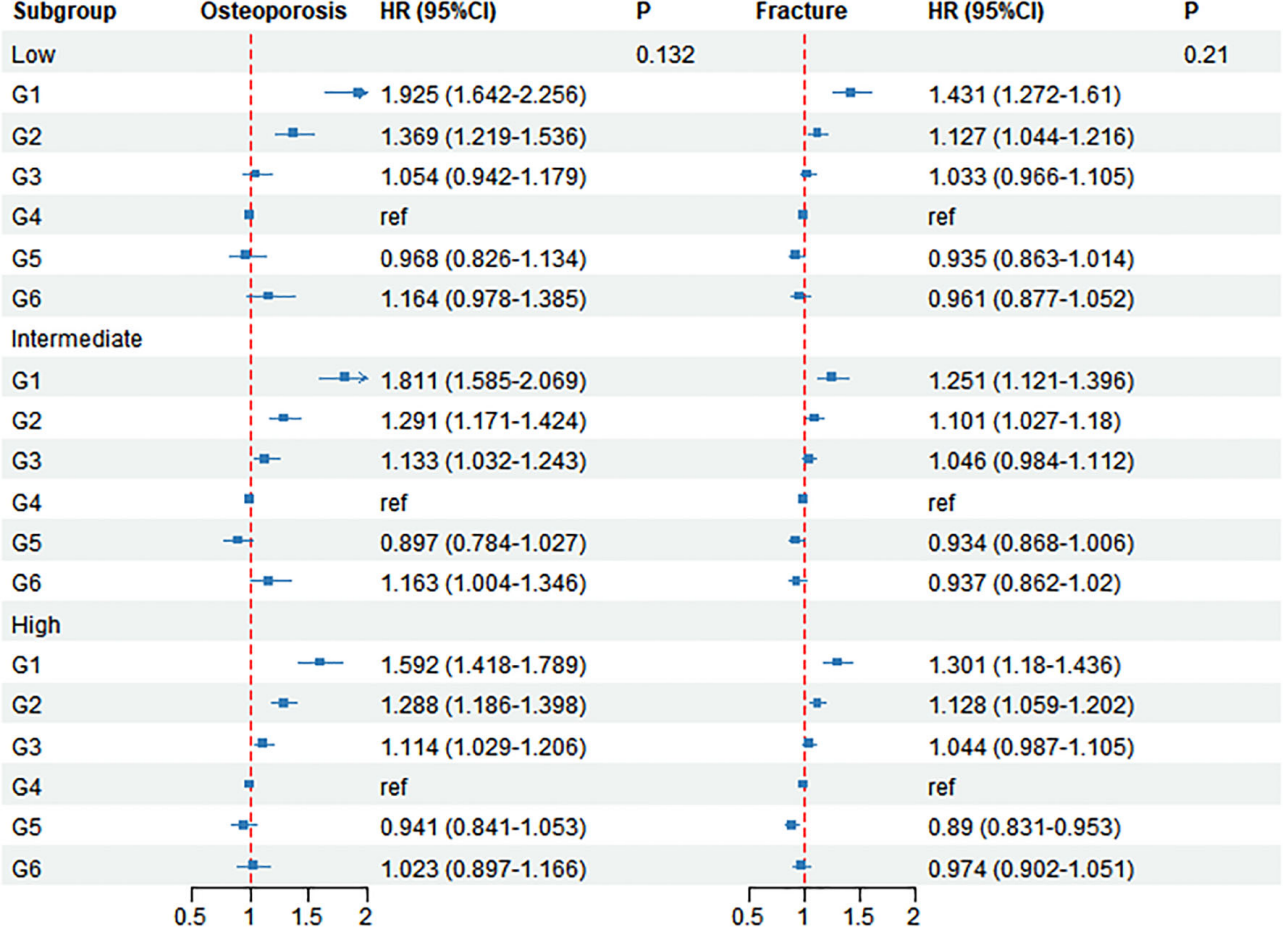
PRS-stratified associations of creatinine with incident osteoporosis and tests for interaction. HR: hazard ratio; CI: confidence interval; PRS: polygenic risk score; ref: reference; G1: group 1; G2: group 2; G3: group 3; G4: group 4; G5: group5; G6: group 6. Forest plot by osteoporosis PRS tertiles (low, intermediate, high) showing higher osteoporosis risk at low creatinine (**G1**) across all tertiles (e.g., HR = 1.925, 1.811, 1.592), with comparable directions for fractures; creatinine×PRS interactions are not significant.

In Cox models stratified according to PRS for osteoporosis (**Figure 4**), the effect of serum creatinine on incident osteoporosis and incident fractures followed a consistent pattern, showing a protective decrease as creatinine increased from G1 (lowest) to G4 (moderate), with an increased risk at higher creatinine levels. In participants with incident osteoporosis, the HR (95% CI) for low creatinine (G1–G3) were as follows: PRS-low: 1.925 (1.642–2.256), 1.369 (1.219–1.536), 1.054 (0.942–1.179); PRS-intermediate: 1.811 (1.585–2.069), 1.291 (1.171–1.424), 1.133 (1.032–1.243); PRS-high: 1.592 (1.418–1.789), 1.288 (1.186–1.398), 1.114 (1.029–1.206); all > 1 (all P < 0.05). In the higher creatinine range, the HR (95% CI) for G5/G6 relative to G4 were as follows: PRS-low: 0.968 (0.826–1.134)/1.164 (0.978–1.385); PRS-intermediate: 0.897 (0.784–1.027)/1.164 (1.004–1.346); PRS-high: 0.941 (0.841–1.053)/1.023 (0.897–1.166).

In the incident fracture group, the HR (95% CI) for G1/G2/G3 were as follows: PRS-low: 1.431 (1.272–1.610), 1.127 (1.044–1.216), 1.033 (0.966–1.106); PRS- intermediate: 1.251 (1.121–1.396), 1.101 (1.027–1.180), 1.046 (0.984–1.112); PRS-high: 1.301 (1.180–1.436), 1.128 (1.059–1.202), 1.044 (0.987–1.105). In the higher creatinine range, the fracture HR for G5/G6 relative to G4 were as follows: PRS-low: 0.935 (0.863–1.014)/0.961 (0.878–1.052); PRS- intermediate: 0.934 (0.868–1.006)/0.937 (0.862–1.020); PRS-high: 0.890 (0.831–0.953)/0.974 (0.902–1.051). The interaction between osteoporosis and creatinine (P = 0.132) and the interaction between incident fractures and creatinine (P = 0.21) were not significant in the global test, indicating that the protective association does not differ substantially across PRS strata.

### The risk of osteoporosis and fracture in different osteoporosis age subgroups

**Figure 5:**
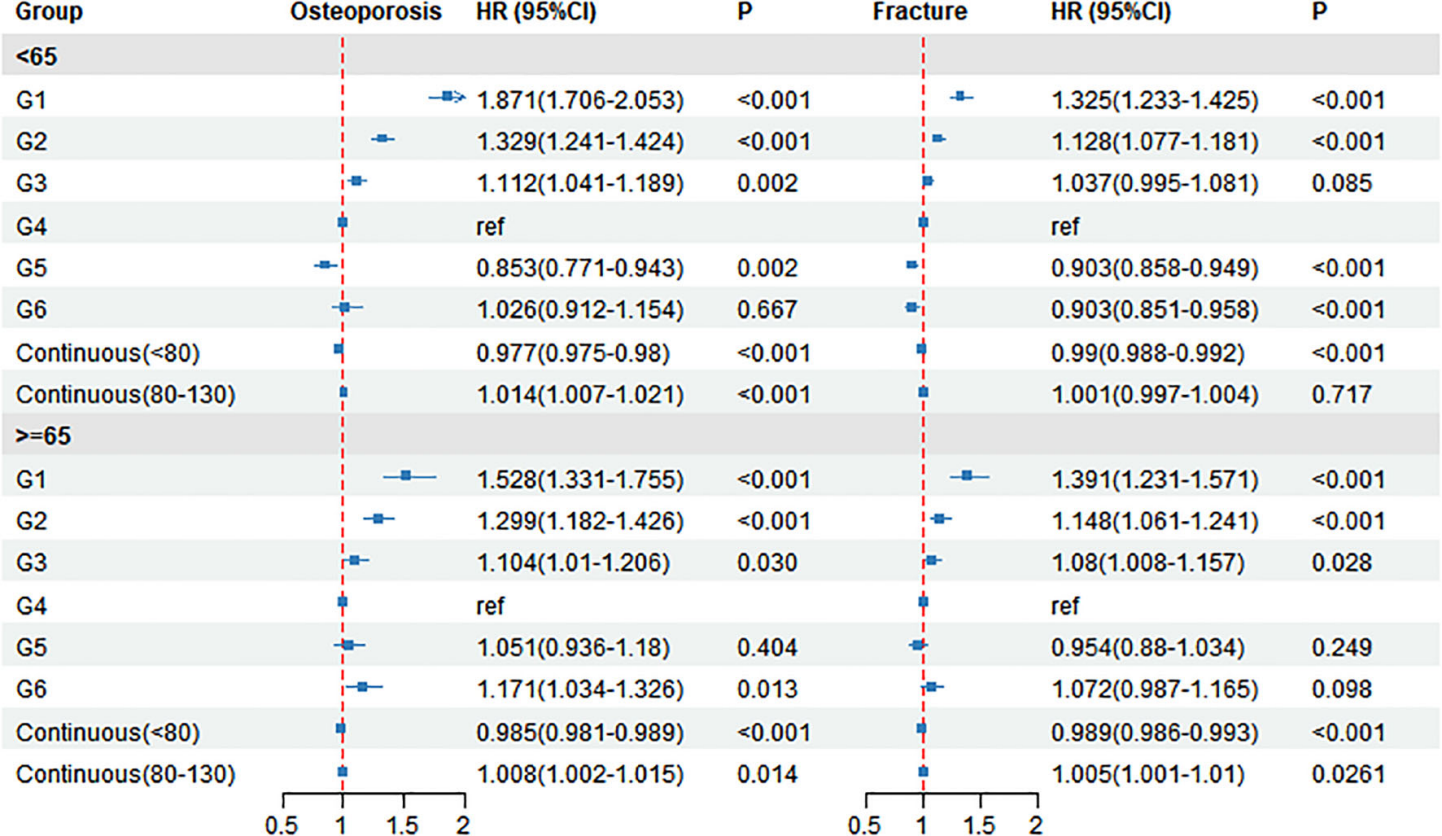
Age-stratified associations between serum creatinine categories and risks of incident osteoporosis and fracture Forest plot of multivariable-adjusted hazard ratios (HRs) and 95% confidence intervals (CIs) for incident osteoporosis and incident fracture across baseline serum creatinine categories, stratified by age (<65 vs ≥65 years). G4 served as the reference category (HR = 1.00). Estimates are shown for categorical groups (G1–G6) and for piecewise continuous creatinine within two ranges (<80 and 80–130 μmol/L). P values correspond to each comparison. The vertical dashed line indicates HR = 1.00.

In Cox regression models stratified by age (<65 vs ≥65; **Figure 5**), the associations between serum creatinine and incident osteoporosis/fractures were evaluated with G4 (70–80 µmol/L) as the reference. For incident osteoporosis in participants aged <65 years, the HRs (95% CIs) for G1–G3 were 1.871 (1.706–2.053), 1.329 (1.241–1.424), and 1.112 (1.041–1.189), respectively (P < 0.001, P < 0.001, and P = 0.002), while the HRs for G5 and G6 were 0.853 (0.771–0.943; P = 0.002) and 1.026 (0.912–1.154; P = 0.667). In the piecewise continuous analysis, each 1 µmol/L increase in creatinine corresponded to HR = 0.977 (95% CI 0.975–0.98; P < 0.001) for <80 µmol/L and HR = 1.014 (95% CI 1.007–1.021; P < 0.001) for 80–130 µmol/L. Among participants aged ≥65 years, the HRs (95% CIs) for G1–G3 were 1.528 (1.331–1.755), 1.299 (1.182–1.426), and 1.104 (1.01–1.206) (P < 0.001, P < 0.001, and P = 0.030), and for G5/G6 were 1.051 (0.936–1.18; P = 0.404) and 1.171 (1.034–1.326; P = 0.013); the corresponding piecewise continuous HRs were 0.985 (0.981–0.989; P < 0.001) for <80 µmol/L and 1.008 (1.002–1.015; P = 0.014) for 80–130 µmol/L.

For incident fractures in participants aged <65 years, the HRs (95% CIs) for G1–G3 were 1.325 (1.233–1.425), 1.128 (1.077–1.181), and 1.037 (0.995–1.081) (P < 0.001, P < 0.001, and P = 0.085), while the HRs for G5 and G6 were 0.903 (0.858–0.949; P < 0.001) and 0.903 (0.851–0.958; P < 0.001). The piecewise continuous HRs were 0.99 (0.988–0.992; P < 0.001) for <80 µmol/L and 1.001 (0.997–1.004; P = 0.717) for 80–130 µmol/L. In participants aged ≥65 years, the HRs (95% CIs) for G1–G3 were 1.391 (1.231–1.571), 1.148 (1.061–1.241), and 1.08 (1.008–1.157) (P < 0.001, P < 0.001, and P = 0.028), and for G5/G6 were 0.954 (0.88–1.034; P = 0.249) and 1.072 (0.987–1.165; P = 0.098); the corresponding piecewise continuous HRs were 0.989 (0.986–0.993; P < 0.001) for <80 µmol/L and 1.005 (1.001–1.01; P = 0.026) for 80–130 µmol/L.

## Discussion

To the best of our knowledge, this is the first population-based prospective cohort study investigating the association between serum creatinine and incident osteoporosis and fractures, while also evaluating its potential interaction with PRS. Using data from 385,576 participants and more than 13 years of median follow-up, we observed a significant U-shaped dose-response relationship between serum creatinine and osteoporosis, and a J-shaped dose-response relationship between serum creatinine and incident fractures. Specifically, in the 25–80 µmol/L range, each 1 µmol/L increase in creatinine was associated with a 2% reduction in osteoporosis risk (P < 0.001) and a 1.0% reduction in fracture risk (P < 0.001); in the 80–130 µmol/L range, each 1 µmol/L increase was associated with a 1.1% increase in osteoporosis risk (P < 0.001) and a slight, non-significant 0.3% increase in fracture risk (P = 0.064). Sex-stratified analyses suggested that the risk nadir may differ by sex (e.g., G5 in men and G4 in women), indicating that a single universal threshold may not apply across sexes. Nevertheless, the overall risk pattern remained broadly consistent. Across PRS tertiles, the associations were directionally consistent and of comparable magnitude, indicating that the physiological–behavioral signal captured by creatinine is robust regardless of underlying genetic risk. Prior research has focused on the interrelations among chronic kidney disease (CKD), osteoporosis, and fractures, as well as clinical management, while the association between skeletal muscle mass and osteoporosis has been relatively understudied. In light of our findings, we propose an integrative framework (**Supplementary Figure S1**) that consolidates the observed non-linear associations between serum creatinine and the risks of osteoporosis and fractures, together with plausible underlying mechanisms. This framework emphasizes the dual role of creatinine as a cross-system biomarker within the muscle–kidney–bone axis and suggests a potential “protective window” at mid-range concentrations (approximately 80 μmol/L), where the associated risk appears to be lowest. Collectively, these insights may enable earlier identification of individuals at elevated risk of osteoporosis and help inform clinical strategies for fracture prevention.

Serum creatinine is more than a surrogate marker of renal function; it is an integrated biomarker shaped by both creatinine generation (muscle mass, dietary intake, and metabolism) and elimination (glomerular filtration, tubular secretion, and transporter-mediated handling). Accordingly, the same creatinine concentration can arise from distinct underlying physiological states. Thus, even after adjustment for BMI and physical activity, the remaining association may reflect aspects of the muscle–kidney axis that are not fully captured by conventional clinical measures. Moreover, proximal tubular secretion is an important component of creatinine elimination. This process is mediated by renal transporters—particularly OCT2 and MATE1/2-K—and can be influenced by disease states or drug–transporter interactions (24). Such influences may increase serum creatinine concentrations without a commensurate reduction in true glomerular filtration rate. This transporter-dependent component offers a biologically plausible explanation for residual unmeasured confounding; for example, diuretics or other medications associated with fracture risk may also affect creatinine handling (25). Collectively, these considerations support viewing creatinine as an integrated phenotype rather than a marker of a single organ system. Osteoporosis is a chronic condition, and its U-shaped association likely reflects two distinct biological pathways. The left branch (i.e., low to moderate creatinine levels) indicates that lower creatinine is associated with a higher risk of osteoporosis. Low creatinine levels are commonly associated with sarcopenia and/or malnutrition (8, 26), with mechanisms such as reduced skeletal loading (27), suppression of myogenic anabolic signaling (e.g., Insulin-like Growth Factor-1 and myokines) (28, 29), and coexisting low-grade inflammation and reduced physical activity (30, 31); these factors are generally linked to lower BMD and increased fracture susceptibility. A fracture is a specific clinical event defined by bone breakage or cracks, influenced by multiple factors such as bone density, bone microstructure, external forces, and bone strength (32), thus exhibiting different patterns in the dose-response relationship, but with an overall similar trend. Mechanistically, the muscle–bone relationship extends beyond purely mechanical coupling. In addition to load-bearing effects, skeletal muscle and bone also function as endocrine organs and communicate bidirectionally through myokines and osteokines that regulate osteoblast and osteoclast activity and coordinate bone remodeling (33).

In multivariate analysis, controlling for covariates such as age, sex, BMI, physical activity, socioeconomic status, and environmental exposures, the risk reduction in multiple categories remained statistically significant (e.g., for osteoporosis, G1 relative to G4: HR = 1.764, 95% CI 1.630–1.894, and for fractures, HR = 1.337, 95% CI 1.248–1.424), indicating that the “sarcopenia–nutrition” pathway is not fully explained by these covariates and may have an independent residual effect. The right branch (higher creatinine levels) suggests that elevated creatinine is linked to a higher risk of osteoporosis. Even below the diagnostic threshold for CKD, elevated creatinine may suggest early GFR decline and disturbances in acid-base and calcium-phosphate homeostasis (34–36), which, through mechanisms such as secondary hyperparathyroidism, decreased active vitamin D, increased fibroblast growth factor 23, and metabolic acidosis (37, 38), result in CKD–MBD-related bone turnover abnormalities and an increased risk of bone loss (39). At the molecular level, dysregulation of the FGF23–Klotho axis is increasingly recognized as a key pathway linking declining kidney function to disrupted phosphate homeostasis, impaired vitamin D activation, and altered parathyroid hormone regulation, ultimately reshaping bone turnover (40). Accordingly, this axis provides a coherent mechanistic link supporting the high-creatinine arm of our proposed model.

It should be noted that high creatinine strata typically have a higher proportion of males and larger BMI, which would conventionally have a protective effect on BMD (41) and theoretically reduce risk; however, we still observe an increased risk, suggesting that at higher creatinine levels, the detrimental effects of kidney function, acid-base balance, and mineral-bone metabolism axis may outweigh potential confounding factors, resulting in a net risk increase. This underscores that creatinine, as a comprehensive marker, reflects both muscle status/fitness and early kidney metabolic dysregulation and is independently associated with the risk of osteoporosis and fractures. Conversely, the curve’s lowest point (lowest risk at moderate creatinine levels) likely represents a state of “adequate muscle and kidney metabolic balance”: the minimum around 80 µmol/L falls within the physiological mid-range, aligning with the integrated phenotype of “sufficient muscle mass, preserved kidney function, optimal mechanical load, stable metabolic/endocrine signals, and improved physical activity.” In the absence of significant kidney damage, slightly elevated creatinine is more likely to reflect better muscle and functional status. Because serum creatinine is determined by both generation and elimination, elevated creatinine at older ages may reflect a composite muscle–kidney phenotype rather than kidney dysfunction alone. Age-related changes in muscle turnover and renal reserve may co-evolve, and thus the relative contribution of muscle versus kidney components may vary across older age brackets.

In age-stratified analyses (<65 vs ≥65 years), we observed a broadly consistent biphasic pattern; however, the location of the apparent risk nadir and the trajectory of the high-creatinine limb shifted with age. For osteoporosis, participants aged <65 years had the lowest risk in a slightly higher creatinine category (G5), with a protective association relative to G4 (G5 vs G4: HR = 0.853, 95% CI 0.771–0.943). In contrast, this apparent benefit was attenuated among those aged ≥65 years (G5 vs G4: HR = 1.051, 95% CI 0.936–1.180), and risk increased significantly in the highest creatinine category (G6 vs G4: HR = 1.171, 95% CI 1.034–1.326). Consistent with the categorical analyses, the inverse association below 80 μmol/L persisted in both age strata but was weaker in older adults. Meanwhile, the positive association above 80 μmol/L remained evident for osteoporosis risk in both age groups when creatinine was modeled continuously within 80–130 μmol/L (HR = 1.014 for <65 years; HR = 1.008 for ≥65 years). For fractures, age-related differences were more pronounced. In the <65-year group, higher creatinine levels (80–130 μmol/L) did not show a clear risk gradient, whereas in the ≥65-year group, creatinine above 80 μmol/L was associated with a modest but statistically significant increase in fracture risk (continuous HR = 1.005; P = 0.026). Collectively, these findings suggest that the “lowest-risk zone” is age dependent and that the clinical implications of higher creatinine differ between younger and older participants. A biologically plausible explanation is that aging reweights the relative contributions of the two principal determinants of serum creatinine—generation and clearance. With advancing age, sarcopenia and declining muscle mass reduce creatinine generation, while renal functional reserve and tubular handling capacity may also deteriorate (42). Consequently, an elevated creatinine concentration in older adults may more specifically reflect impaired renal reserve and early disturbances in mineral and acid–base metabolism (even before overt chronic kidney disease becomes clinically apparent), thereby strengthening the risk gradient on the high-creatinine limb. By contrast, in younger adults with relatively preserved renal handling, higher creatinine is more likely to indicate greater muscle mass and better physical fitness (43), which could explain why the nadir occurs at a higher creatinine category (G5) and why no obvious fracture-risk gradient is observed above 80 μmol/L. The attenuation of the inverse association below 80 μmol/L in older adults may additionally reflect greater etiologic heterogeneity underlying low creatinine (e.g., varying contributions from sarcopenia, multimorbidity, and frailty), as well as the increasing importance of non-skeletal determinants of fractures—such as falls and neuromuscular dysfunction—in later life. Taken together, these results support an age-specific interpretation of creatinine-based risk stratification and highlight the need for future studies integrating direct muscle phenotyping (e.g., grip strength and lean mass) and alternative renal biomarkers (e.g., cystatin C–based eGFR) to disentangle the respective contributions of muscle and kidney function and to refine age-appropriate “lowest-risk” ranges.

In the sex-stratified subgroup analysis, the dose–response confirmed a similar biphasic association in men and women: as creatinine increased, risk declined across the low-to-middle range and then rose at higher levels, with the nadir and its range differing between the sexes. In men, the lowest risk was observed at G5 (G5 relative to G4 for osteoporosis: HR = 0.837, 95% CI 0.730–0.949; fracture: HR = 0.899, 95% CI 0.858–0.950), whereas in women, it was at G4 (no significant difference between G5 and G4, osteoporosis: HR = 1.075, 95% CI 0.960–1.192; fracture: HR = 1.042, 95% CI 0.959–1.118, P > 0.05). This suggests that the creatinine range corresponding to the “nadir” of risk in men is higher than in women, consistent with sex differences in creatinine distribution (kernel density plot: women generally lower, men higher). In line with a large US study, serum creatinine in both women and men increases with age (women approximately 0.88–1.10 mg/dL; men approximately 1.00–1.29 mg/dL) (44), a pattern that corresponds with the lower osteoporosis incidence in men, or suggests that higher creatinine could be a proxy for greater muscle mass, though causality cannot be inferred. In the higher creatinine range, men with osteoporosis in G6 relative to G4 showed no significant difference (HR = 0.958, 95% CI 0.860–1.064, P = 0.503), while fractures showed a notable association (HR = 0.927, 95% CI 0.880–0.971, P = 0.007), whereas in women, the risk significantly increased (osteoporosis G6 relative to G4: HR = 1.401, 95% CI 1.242–1.582; fracture: HR = 1.249, 95% CI 1.111–1.388, both P < 0.05). Given the differences in absolute creatinine levels and distribution between sex, direct comparisons at the same threshold should be approached with caution; therefore, we emphasize patterns “within the same sex” rather than asserting a stronger effect in one sex. Specifically, men exhibited a more significant decrease in the 25–80 µmol/L range (each 1 µmol/L: HR = 0.954, 95% CI 0.945–0.960, P < 0.05), while the increase in the 80–130 µmol/L range was slower and non-significant (HR = 1.005, 95% CI 0.997–1.012; P = 0.198), consistent with the explanation that the “sarcopenia–nutrition” pathway has a larger effect. The lowest risk in women was observed at G4, and the increase in the higher creatinine range was steeper (for osteoporosis, 80–130 µmol/L, each 1 µmol/L: HR = 1.017, 95% CI 1.011–1.024; for fractures, HR = 1.007, 95% CI 1.003–1.014). The left branch is relatively flatter (25–80 µmol/L range, each 1 µmol/L for osteoporosis: HR = 0.982, 95% CI 0.979–0.985; for fractures: HR = 0.992, 95% CI 0.990–0.994), suggesting that women may be more sensitive to the kidney-mineral/acid-base axis, which may better capture osteoporosis risk.

Osteoporosis is a complex, highly heritable, polygenic disease (45). During follow-up, osteoporosis PRS was significantly associated with the risk of osteoporosis (17, 18). Previous research has reported a positive correlation between bone mass and behaviors (e.g., calcium intake, lower-fat dietary patterns, higher physical activity), and suggested interactions with genetic variations related to bone mass (46–48). We found no evidence that genetic risk modifies the protective association between serum creatinine and incident osteoporosis (interaction test: osteoporosis P = 0.132; incident fractures P = 0.21). PRS mainly reflects baseline bone metabolic characteristics (e.g., trabecular structure and bone formation/absorption tendencies), representing lifelong genetic susceptibility (49, 50); creatinine should be considered a “current physiological phenotype”—a readout that integrates muscle mass and kidney metabolic status—sensitive to lifestyle, nutrition, and the spectrum of chronic diseases (51, 52). As a result, genetic burden did not substantially alter the environmental and physiological signals encoded by creatinine. Therefore, this “protective window” remains significant across varying levels of genetic risk, highlighting both its universality and potential for intervention: improving muscle strength, nutrition, and regulating the kidney-mineral metabolism axis could collectively “pull” individuals away from the high-risk areas at both ends of the nonlinear curve.

This study offers several strengths: the large sample size and long-term follow-up helped to more accurately characterize the nonlinear pattern, covering both ends of the distribution; using a unified analysis platform, we comprehensively adjusted for demographic, lifestyle, anthropometric/metabolic, socioeconomic, and environmental factors, with consistent results across genetic risk strata; the combined use of restricted cubic splines and classification models provided mutual validation, enhancing robustness and interpretability. However, there are several limitations to consider: First, reliance on a single baseline creatinine measurement, together with the inclusion of potentially traumatic fractures, may lead to regression dilution and bias the estimated associations toward the null. Second, medication use was not comprehensively captured for all participants (e.g., systemic corticosteroids, proton pump inhibitors, diuretics, and vitamin D/calcium supplementation). These exposures may contribute to residual confounding, potentially with heterogeneous directions of bias. For example, corticosteroids can adversely affect both skeletal muscle and bone, which may preferentially elevate risk at the low-creatinine end; diuretics can influence volume status and serum creatinine concentrations and have also been associated with falls and fracture risk; and vitamin D supplementation may reflect confounding by indication rather than a causal effect. Accordingly, this potential bias should be considered when interpreting the observed associations. Third, despite long-term follow-up and exclusion/adjustment of early events, reverse causality cannot be completely excluded. Fourth, in women, menopausal status and estrogen therapy are major determinants of bone loss and may also influence serum creatinine through changes in body composition. Although we adjusted for age, the absence of detailed information on hormonal status and circulating sex hormone levels may result in residual confounding, particularly in sex-stratified analyses. While serum creatinine may serve as a pragmatic indicator of the muscle–kidney milieu, future studies should incorporate direct assessments of muscle mass and function (e.g., handgrip strength and lean mass) to more explicitly test muscle-mediated pathways, disentangle the respective contributions of muscle and kidney function to the observed non-linear patterns, and evaluate the incremental predictive utility of creatinine beyond established tools such as DXA and FRAX and clinically actionable thresholds.

## Conclusion

Taken together, serum creatinine demonstrates a nonlinear, biphasic relationship with incident osteoporosis: increases within the low-to-intermediate range likely reflect greater muscle strength and nutritional status and are associated with lower risk, whereas higher-level elevations are more likely to signal early kidney–mineral axis dysfunction and are associated with increased risk. This pattern was consistent across polygenic-risk strata. Given its low cost, ready availability, and amenability to intervention, serum creatinine merits consideration as a practical adjunct to osteoporosis risk stratification and longitudinal follow-up.

## Data Availability

Publicly available datasets were analyzed in this study. This data can be found here: https://biobank.ctsu.ox.ac.uk/(85224).
