# Supplementary material for: Serum creatinine, genetic susceptibility, and the risk of osteoporosis and fracture: a prospective cohort study from the UK Biobank
Source: Front Endocrinol (Lausanne). 2026 Jan 20;16:1727636. doi: 10.3389/fendo.2025.1727636 (PMC12864067; doi:10.3389/fendo.2025.1727636)
Supplement: Supplementary file 1 [file DataSheet1.docx]

***Supplemental Digital Content***

**Serum Creatinine, Genetic Susceptibility, and the Risk of Osteoporosis and Fracture: A Prospective Cohort Study from the UK Biobank**

**Journal name：Osteoporosis International**

**Author names:** Weijie He; Yu Zhou; Jiaxuan Ding; Jiale Jiang; Chonghui Hu ; Qiongyan Liu ; Honglin Gu; Huimou Chen

**Corresponding author:**

**Huimou Chen (Last Corresponding Author)**

Department of Oncology, Sun Yat-sen Memorial Hospital, Sun Yat-sen University, Guangzhou, Guangdong 510120, P.R. China

Email: chenhm88@mail.sysu.edu.cn

**Honglin Gu,**

Department of Spine Surgery, Guangdong Provincial People's Hospital (Guangdong Academy of Medical Sciences), Southern Medical University, No. 106 Zhongshan Road II, Guangzhou 510080, Guangdong Province, China

Email: guhonglin@gdph.org.cn

**Qiongyan Liu**

**P**hase I Clinical Trial Centre, Sun Yat-sen MemorialHospital, Sun Yat-sen University. Guangzhou, Guangdong 510655, P.R. China.

Email: liuyq46@mail.sysu.edu.cn

**Supplementary Table S1 Data field and description of basic information in UK Biobank**

**Supplementary Table S2** **ICD-10 and ICD-9 codes for diseases.**

**Supplementary Table S3** **Baseline Characteristics of the Study Population Stratified by creatines.**

**Supplementary Figure S1 Conceptual model of nonlinear associations between serum creatinine and risks of osteoporosis and fracture**

**Supplementary Table S1 Data field and description of basic information in UK Biobank**

| **Basic information** | **Data field** | **Description** |
| --- | --- | --- |
| Age | 21022 | Age at recruitment |
| Sex | 31 | Sex at recruitment |
| Townsend | 22189 | Townsend deprivation index at recruitment |
| Ethnic | 21000 | Ethnic background |
| BMI | 21001 | Body mass index (BMI) |
| Alcohol intake frequency | 1558 | Alcohol intake frequency |
| Smoking | 20116 | Smoking status |
| Qualification | 6138 | Category of qualification |
| Sleep duration | 1160 | Sleep duration in every 24 hours |
| Physical activity | 22036 | At or above moderate/vigorous/walking recommendation |
| PM2.5 | 24006 | Particulate matter air pollution (pm2.5) |
| Date of death | 40000 | Date of death |
| Diagnoses - ICD10 | 41270 | Hospital inpatient |
| Diagnoses - ICD9 | 41271 | Hospital inpatient |
| osteoporosis with pathological fracture | 131962 | Date M80 first reported (osteoporosis with pathological fracture) |
| osteoporosis without pathological fracture | 131964 | Date M81 first reported (osteoporosis without pathological fracture) |
| osteoporosis in diseases classified elsewhere | 131966 | Date M82 first reported (osteoporosis in diseases classified elsewhere) |
| Fracture | 2463 | Fractured/broken bones in last 5 years |
| chronic renal failure | 132032 | Date N18 first reported (chronic renal failure) |
| Health outcomes + Self-reported medical conditions | 20002 | Non-cancer illness code, self-reported |
| Creatinine | 30700 | Creatinine |
| PRS | 26258 | Standard PRS for osteoporosis |

**Supplementary Table S2** **ICD-10 and ICD-9 codes for diseases.**

| **Disease at or before baseline** | **ICD-10 code** | **ICD-9 code** |
| --- | --- | --- |
| Osteoporosis | M80, M81, M82 | 7330 |
| Fracture | M907\|S02\|S12\|S22\|S32\|S42\|S52\|S62\|S72\|S82\|S92\|T02\|T12 | 800-829,7331 |
| Disorders of continuity of bone | M84 |  |
| Osteopathies, chondropathies, and acquired musculoskeletal deformities |  | 730-739 |
| Chronic kidney disease | N18 | 585 |

**Supplementary Table S3 Baseline Characteristics of the Study Population Stratified by creatines**

|  | G1 | G2 | G3 | G4 | G5 | G6 | p test |
| --- | --- | --- | --- | --- | --- | --- | --- |
| n | 15436 | 76779 | 119525 | 106219 | 69140 | 47798 |  |
| Ethic category = White (%) | 13454 (87.16) | 69025 (89.90) | 108764 (91.00) | 97053 (91.37) | 63430 (91.74) | 43551 (91.11) | <0.001 |
| Alcohol category (%) |  |  |  |  |  |  | <0.001 |
| <3 times/week | 7858 (50.91) | 39424 (51.35) | 59950 (50.16) | 49462 (46.57) | 31188 (45.11) | 22381 (46.82) |  |
| >=3 times/week | 5734 (37.15) | 30342 (39.52) | 50214 (42.01) | 49359 (46.47) | 33716 (48.76) | 21844 (45.70) |  |
| Never/Other | 1844 (11.95) | 7013 ( 9.13) | 9361 ( 7.83) | 7398 ( 6.96) | 4236 ( 6.13) | 3573 ( 7.48) |  |
| Smoking category (%) |  |  |  |  |  |  | <0.001 |
| Current | 2088 (13.53) | 8312 (10.83) | 12647 (10.58) | 11478 (10.81) | 6735 ( 9.74) | 4082 ( 8.54) |  |
| Never | 8573 (55.54) | 44008 (57.32) | 67055 (56.10) | 56927 (53.59) | 36950 (53.44) | 25061 (52.43) |  |
| Previous | 4775 (30.93) | 24459 (31.86) | 39823 (33.32) | 37814 (35.60) | 25455 (36.82) | 18655 (39.03) |  |
| BMI category (%) |  |  |  |  |  |  | <0.001 |
| >30 | 3534 (22.89) | 16547 (21.55) | 27512 (23.02) | 26291 (24.75) | 17875 (25.85) | 14256 (29.83) |  |
| 25-30 | 5141 (33.31) | 27192 (35.42) | 47097 (39.40) | 47812 (45.01) | 34333 (49.66) | 24341 (50.92) |  |
| Other | 6761 (43.80) | 33040 (43.03) | 44916 (37.58) | 32116 (30.24) | 16932 (24.49) | 9201 (19.25) |  |
| Physical category = Other (%) | 6247 (40.47) | 29277 (38.13) | 43622 (36.50) | 36966 (34.80) | 23311 (33.72) | 17178 (35.94) | <0.001 |
| sex = 1 (%) | 402 ( 2.60) | 5309 ( 6.91) | 30327 (25.37) | 64725 (60.94) | 57653 (83.39) | 42836 (89.62) | <0.001 |
| Qualification category (%) |  |  |  |  |  |  | <0.001 |
| High | 4933 (31.96) | 25255 (32.89) | 39594 (33.13) | 35713 (33.62) | 23187 (33.54) | 15005 (31.39) |  |
| Intermediate | 6873 (44.53) | 34835 (45.37) | 54153 (45.31) | 47511 (44.73) | 30887 (44.67) | 21042 (44.02) |  |
| Low | 3630 (23.52) | 16689 (21.74) | 25778 (21.57) | 22995 (21.65) | 15066 (21.79) | 11751 (24.58) |  |
| Townsend (mean (SD)) | -1.08 (3.16) | -1.29 (3.07) | -1.34 (3.04) | -1.37 (3.05) | -1.46 (3.04) | -1.36 (3.11) | <0.001 |
| Sleep duration (mean (SD)) | 7.08 (1.34) | 7.11 (1.28) | 7.11 (1.25) | 7.11 (1.23) | 7.10 (1.20) | 7.15 (1.27) | <0.001 |
| Age (mean (SD)) | 55.95 (7.97) | 55.74 (8.00) | 55.94 (8.03) | 56.36 (8.09) | 56.76 (8.14) | 58.09 (8.06) | <0.001 |
| PM_25 (mean (SD)) | 10.05 (1.03) | 10.00 (1.01) | 9.98 (1.01) | 9.97 (1.00) | 9.95 (1.00) | 9.97 (1.02) | <0.001 |

| outcome | biomarker | analysis_range | hr | lower_ci | upper_ci | p_value | n_obs |
| --- | --- | --- | --- | --- | --- | --- | --- |
| OSTE event | creat | 0 to 80 | 0.980127 | 0.978085 | 0.982174 | 2.56E-79 | 317959 |
| OSTE event | creat | 80 to 130 | 1.012348 | 1.007963 | 1.016752 | 3.00E-08 | 116006 |

**Supplementary Figure S 1 Conceptual model of nonlinear associations between serum creatinine and risks of osteoporosis and fracture**


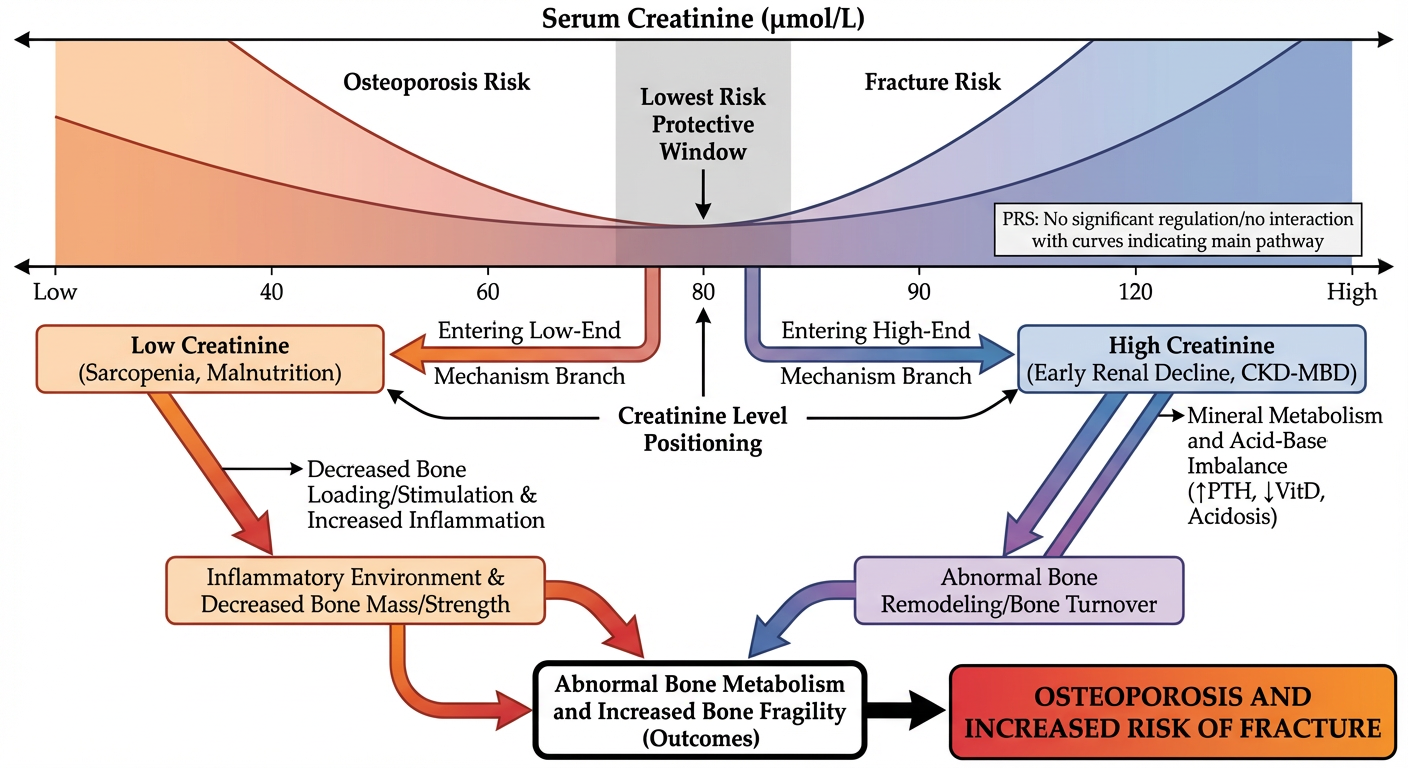


This schematic summarizes the observed nonlinear dose–response patterns between baseline serum creatinine and incident osteoporosis (U-shaped) and fractures (J-shaped), highlighting a lowest-risk zone in the mid-range (approximately 80 μmol/L). The left branch illustrates potential pathways linked to low creatinine (e.g., sarcopenia and/or malnutrition) leading to reduced mechanical loading and a pro-inflammatory milieu with decreased bone mass/strength. The right branch depicts potential pathways linked to high creatinine suggestive of early renal functional decline and CKD–mineral and bone disorder (CKD-MBD), including disturbances in mineral and acid–base homeostasis that may contribute to abnormal bone remodeling/turnover. PRS showed no significant interaction with the creatinine–outcome curves in this study. PRS, polygenic risk score; CKD-MBD, chronic kidney disease–mineral and bone disorder; PTH, parathyroid hormone; VitD, vitamin D.
